# Heat pretreatment of canine samples to evaluate efficacy of imidacloprid + moxidectin and doxycycline in heartworm treatment

**DOI:** 10.1186/s13071-017-2189-2

**Published:** 2017-05-19

**Authors:** Alexandre José Rodrigues Bendas, Flavya Mendes-de-Almeida, Cristiano Von Simson, Norma Labarthe

**Affiliations:** 1 Programa de Pós-Graduação em Medicina Veterinária, Faculdade de Veterinária Universidade Federal Fluminense, R. Vital Brazil Filho, 64, Santa Rosa, Niterói-RJ 24230-340 Brazil; 2 Veterinary Services, Virbac US P.O. box 162059, Ft Worth, TX 76161 USA; 3 Fundação Oswaldo Cruz, Av. Brasil, 4365, Rio de Janeiro, RJ 21040-900 Brazil

**Keywords:** *Dirofilaria immitis*, Heartworm, Adulticide treatment, Antigen detection

## Abstract

**Background:**

Considering the recent information on the increase of *Dirofilaria immitis* antigen detection by rapid assays in canine blood samples after heat treatment, the proposal that immune complexes block *D. immitis* antigen detection and that macrocyclic lactone + doxycycline (alternative protocol) might lead to increased production of those immune complexes, resulting in the erroneous diagnosis of adult worm elimination, and that there is no recommended adulticide marketed in Brazil, a study was performed to evaluate the interference of moxidectin + doxycycline (moxi-doxy) on diagnostic procedures when heartworm positive dogs are treated with this alternative protocol. Twenty-two naturally infected pet dogs were treated monthly with topical 10% imidacloprid + 2.5% moxidectin and with oral doxycycline (10 mg/kg BID/30 days) (moxi-doxy). All the dogs had their microfilaremia level determined prior to the first day of treatment, and were tested every 6 months for microfilariae (Mf) detection prior to heating, and for antigen detection prior to and after heating, the sample.

**Results:**

The results indicate that the treatment protocol can eliminate adult heartworms as early as 6 months after the first dose, especially in low microfilaremic dogs (< 300 Mf/ml). In this study, all dogs were free of heartworm antigen after 18–24 months of treatment. In a comparison of pre-heated samples and non-heated samples, sample pre-heating increased antigen detection sensitivity, and non-heated samples tended to be antigen-negative earlier than the pre-heated samples, especially when dogs had low microfilaremia levels. These discrepancies were not present in a subsequent sample of the same dog 6 months later.

**Conclusions:**

Two negative antigen test results 6 months apart can be recommended as the criterion to consider when a dog has been cleared of infection. The initial microfilaremia level of a dog can be used to estimate the necessary time frame to end the treatment period.

## Background

In Brazil, the prevalence of canine *Dirofilaria immitis* patent infections approaches 62% in some areas [1]. Many owners of infected adult dogs seek treatment for these infections at local clinical practices. Although melarsomine dihydrochloride (Immiticide®, Merial or Diroban®, Zoetis), the internationally recommended treatment for such infections, was once available in Brazil, it is no longer marketed or registered. As the only option of adulticide treatment for dogs, a monthly macrocyclic lactone preventive associated with doxycycline [2], is largely used, even though it has been discouraged within guidelines [2, 3]. An alternative protocol (macrocyclic lactone + doxycycline) therapy is usually discouraged because it may take many months for adult worms to die, allowing the disease to progress during treatment [4], and there are macrocyclic lactone-resistant heartworm development concerns [5, 6].

Rapid antigen detection assays are the tests most practitioners rely upon for the clinical diagnosis of heartworm infections because they were believed to be highly sensitive (95.5–97% of sensitivity) and specific (100% specificity) [7]. In clinical practices, the canine alternative protocol efficacy is also monitored by antigen testing, even though the test results appearing in earlier publications on the adulticidal effects of ivermectin have highlighted the interference of the alternative protocol [8]. In the years following, samples of dogs that had been treated by general practitioners with monthly macrocyclic lactone chemoprophylactic medication and doxycycline were tested to compare antigen detection results before and after heat pre-treatment. This study suggested that the alternative protocol interferes with antigen test results, even though more than half of the dogs included had received an incomplete alternative protocol treatment with different drugs and different doxycycline regimens [9].

Heat pre-treatment of samples has been proven to unbind the blocked antigen in different research scenarios, and an increased antigen detection has been confirmed after heating [10]. The most accepted mechanism to explain this heat treatment effect is through the disruption of antigen-sequestering immune complexes that may be caused by heating. Although an increase in antibody production by the hosts is considered likely, a decrease in the antigen production due to a reduction of their worm burden after treatment cannot be discarded. Therefore, additional data on blocking positive antigen results are needed to explain the effects of adulticidal treatment, with either alternative protocols or melarsomine dihydrochloride treatments, as the mechanism by which heat pre-treating samples enhances antigen detection is still to be completely elucidated [9, 11–14]. To determine how the antigen tests blockage was induced during alternative protocol therapy in clinical settings, pet dogs were submitted to controlled moxi-doxy treatment, and their samples were evaluated before and after heat pre-treatment to detect the elimination of the adult heartworms.

## Methods

### Dogs

All the dogs that participated in this study were referred to the Instituto de Especialidades em Medicina Veterinária (IEMEV), Rio de Janeiro, Brazil, due to a presumptive or confirmed diagnosis of heartworm infection. Consequently, we could not estimate the infection date or worm ages at enrollment. To be included in the study, after the owner’s agreement and consent, a blood sample was collected from each pet dog, and their infection was confirmed by *D. immitis* antigen testing according to manufacturer´s instructions (Snap 4Dx Plus Test, IDEXX Labs, Maine/USA) on day 0. The use of macrocyclic lactones or doxycycline during the prior 6 months was an exclusion criterion.

We tested 22 samples, all of which were positive for *D. immitis* antigen. The dog breeds included one Basset Hound, one Beagle, one Dachshund, two Golden Retrievers, one Labrador Retriever, three German Shepherds, one Miniature Pinscher, two Pugs, and ten mixed breeds. Eight dogs were males, and fourteen were females, with ages between 1 and 8 years and weights between 2.7 kg and 47.8 kg.

### Alternative protocol

Prior to beginning the moxi-doxy treatment (day 0), each dog had an aliquot of the whole blood sample tested for antigen detection (Snap 4Dx Plus Test, IDEXX Labs), microfilariae detection by Knott’s modified test [15], and counting [16].

On day 0, all dogs were administered a monthly topical 10% imidacloprid + 2.5% moxidectin (Advocate®, Lab. Bayer, São Paulo, Brazil), which was repeated every month by the same attending veterinarian. On the same day, all dogs were administered oral doxycycline hyclate at the dose of 10 mg/kg BID/30 days (Doxifin®, Lab. Ourofino, São Paulo, Brazil) by their owners and that was repeated every 6 months. All dogs were released from the study after they were amicrofilaremic and presented two consecutive negative antigen tests results with pre-heated samples.

### Sample testing

Modified Knott’s test [15] was performed every 6 months. Microfilariae counting was performed only on day 0. For microfilariae counting, two slides of each whole blood sample were prepared by spreading a mixture of the sample (20 μl) and water (5 μl) over a 375 mm^2^ area. Slides were dried, Giemsa-stained, and the microfilariae of each slide were counted by two independent technicians. The final microfilariae number was the mean of the four counting results. When no microfilaria could be located on the counting slides, and yet the sample showed microfilariae positive on Knott’s test, the microfilaremia was assumed to be less than 300 Mf/ml [17].

Antigen testing was performed every six months according to manufacturer’s instructions, and when the result was negative, 450 μl of the sample was heated to 104 °C for 10 min, and the resulting coagulum centrifuged [12]. The supernatant was removed and tested according to the manufacturer’s instructions.

## Results

A total of 22 pet dogs, naturally infected with heartworms, met the inclusion and exclusion criteria for enrollment. The dogs were aged from 1 to 8 years, and there were 14 females and 8 males.

At 6 months from the initiation of treatment, the antigen testing results of 14 of the 22 dogs were negative, and 6 of those tested positive after heat pre-treating of the samples. At 12 months, the results for these 6 dogs were before and after heating, and thus, they were released from further testing. At 12 months, 6 more dogs tested antigen-negative before and after heating, and 1 dog tested negative before heating and positive after heating. At 18 months, these 7 dogs tested negative before and after heating, and one additional dog also tested negative before and after heating. Therefore, all 8 of these dogs were released from further testing. The last 2 dogs tested antigen-negative at 18 and 24 months, and they showed no difference in results between their heated and non-heated samples (Table 1).

**Table 1:** The number of *Dirofilaria immitis* naturally infected dogs submitted to a moxi-doxy therapy (10% imidacloprid + 2.5% moxidectin and doxycycline hyclate) that remained antigen-negative even after a heat pre-treatment of samples, according to the microfilariae (Mf) level

Mf level	*n*	Months of moxi-doxy		
		6	12	18
Amicrofilaremic	2	1	1	–
< 300 Mf/ml	9	6^a^	3	–
> 300 Mf/ml	11	1^b^	8	2
Total	22	8	12	2

Different letters (a and b) in column indicate significant differences: *χ* ^2^ = 4.90, *P* = 0.0166

The highest proportion of negative antigen rapid test results that converted to positive test results after heating was observed at 6 months of treatment (42.9%; *P* < 0.01). All dogs testing negative before and after sample heating remained negative in subsequent six-month interval testing (Table 2).

**Table 2:** The number of unheated (UHt) negative *Dirofilaria immitis* antigen (Ag) test results from dogs submitted to a moxi-doxy therapy (10% imidacloprid + 2.5% moxidectin and doxycycline hyclate) that became Ag positive after a sample heat (Ht) pre-treatment

	Months of moxi-doxy			
	6	12	18	24
UHt Ag negative	14	21	14	2
First Ht Ag Negative	8	12	2	–
Second Ht Ag Negative^a^	–	8	12	2
Total Ht Ag positive/ total UHt Ag negative (%)	6/14 (42.9%)^a^	1/21(4.8%)^b^	0/14 (–)	0/2 (–)

^a^Dogs were released from the study after 2 negative pre-heated antigen tests

Different letters (a and b) in line indicate significant differences: *χ* ^2^ = 5.42; *P* = 0.0099

Before the moxi-doxy treatment was initiated, most dogs were microfilaremic at Knott’s test (20/22; 90.9%). Because 8 of their samples showed no microfilariae on counting slides, they were considered to have less than 300 Mf/ml of blood. Another dog’s Mf count was below 300 Mf/ml as well, and therefore, the number of low microfilaremic dogs was assumed to be 9 [17]. A total of 11 dogs were in the higher microfilariae count group, with a total microfilariae count ranging from 438 to 44,538 Mf/ml of blood. After 6 months of treatment, all dogs, regardless of their initial microfilariae count, became amicrofilaremic (Knott’s test) and remained amicrofilaremic until they were released.

Most dogs with lower microfilaremia levels (< 300 Mf/ml of blood) (66.7%) tended to present negative antigen test results (prior to and after heat pre-treatment of samples) faster than the higher microfilaremic dogs (> 300 Mf/ml of blood) (9.1%) (*P* < 0.05). At 12 months of moxi-doxy treatment, all lower microfilaremic dogs, and 72.7% of higher microfilaremic dogs, were found to be antigen-negative before and after heat pre-treatment. At 18 months of treatment, the last 2 higher microfilaremic dogs were found to be antigen-negative before and after heat pre-treatment (Table 2). These 2 dogs were monitored until 24 months after the initiation of treatment, and, when they presented negative antigen results again, they were released from the study.

## Discussion

The results seemed to indicate that the topical imidacloprid + moxidectin and doxycycline (moxi-doxy) treatment can block transmission of heartworms by eliminating the microfilaremia. This treatment begins to eliminate adult heartworms (based on antigen and microfilaria testing) as early as 6 months into the protocol, and, in this study, it achieved *D. immitis* adulticide results (judged by antigen elimination - 2 consecutive pre-heated negative results) by 24 months at the latest.

As the adult worms die, the concentration of circulating antigen in these dogs declines gradually until it eventually falls below test detection limits. Part of the circulating antigen appears to be bound to the antibodies in many (or all) dogs, and thus, heating samples before testing can free this antibody-bound antigen [10]. In addition to freeing this antigen, the process of coagulating the proteins in the plasma sample and then collecting the liquid phase to be used in the test may also concentrate the (carbohydrate) worm antigen in the sample [18]. This would explain why some of the samples that initially tested negative later tested positive after the heating process. The freed antigen and the concentration of the sample would raise them above the detection limit again, for a period of time. Therefore, it is suggested that the immune complexes formation is a natural event during the course of a heartworm infection and that an antigen remnant is detected in non-heated samples. On the other hand, worms from ivermectin-treated dogs are thought to produce less antigen as the treatment progresses [8], and the same could be predicted to occur during the treatment with moxidectin + imidacloprid and doxycycline at 3 months of treatment [19].

As subsequent samples of these same dogs tested negative before and after heating in a consistent manner, we believe that these dogs had no more adult heartworms producing antigen. Therefore, the first negative samples (before heating) were already indicative of success of the adulticide treatment.

It would be very interesting to test successive samples of heartworm-positive dogs, treated with the recommended melarsomine dihydrochloride adulticide protocol, and then observe if the first negative samples would test positive if pre-heated. If this was done, we could then compare the number of cases where the results diverge between non-heated and pre-heated samples, following these two different adulticide protocols.

Most of the dogs that seroconverted to antigen-negative, both before and after the sample heat-treatment, had less than 300 Mf/ml of blood at 6 months of treatment, and the higher microfilariae-level dogs tended to seroconvert at either 12 months of treatment or even 18 months (2 dogs) of treatment. In addition to the fact that dogs with lower Mf counts might have less antigen-producing worms that need to be eliminated, these results also seem to confirm that the presence of microfilariae correlates with a lower immune response towards the parasites. This result has been shown before [20], suggesting that a low microfilariae burden might favor treatment efficacy due to the better immune response towards the adult parasites. Therefore, we suggest that microfilaremia levels may be used by companion animal practitioners to estimate the speed of results with this adulticide treatment. Furthermore, it must be emphasized that once a dog has been infected by heartworms, reinfection is likely to occur. Therefore, practitioners must properly educate their clients, and continuous chemoprophylaxis must be enforced.

## Conclusions

Two negative antigen rapid test results that came 6 months apart might be recommended as the criterion to consider a dog cleared of infection. Furthermore, a dog’s initial microfilaremia level may be used to estimate the necessary time frame to end the treatment period.

## Abbreviations

Mf: Microfilariae
Moxi-doxy: Alternative adulticide protocol using imidacloprid + moxidectin associated with doxycycline
